# MRI Based Fiber Strain Mapping of the Medial Gastrocnemius Muscle at Submaximal Isometric Contractions at Different Ankle Angles

**DOI:** 10.21203/rs.3.rs-2548171/v1

**Published:** 2023-02-09

**Authors:** Brandon Cunnane, Usha Sinha, Vadim Malis, Ryan Hernandez, Edward Smitaman, Shantanu Sinha

**Affiliations:** San Diego State University; San Diego State University; University of California, San Diego; San Diego State University; University of California, San Diego; University of California, San Diego

## Abstract

Muscle force production is influenced by muscle fiber and aponeurosis architecture. This prospective cohort study utilizes special MR imaging sequences to examine the structure-function *in-vivo* in the Medial Gastrocnemius (MG) at three-ankle angles (dorsiflexion, neutral, and plantar flexion) and two submaximal levels of maximum voluntary contraction (25% and 50% MVC). The study was performed on 6 young male subjects. Muscle fiber and aponeurosis strain, fiber strain normalized to force, fiber length and pennation angle (at rest and peak contraction) were analyzed for statistical differences between ankle positions and %MVC. A two-way repeated measures ANOVA and post hoc Bonferroni-adjusted tests were conducted for normal data. A related samples test with Friedman’s 2-way ANOVA by ranks with corrections for multiple comparisons was conducted for non-normal data. The dorsiflexed ankle position generated significantly higher force with lower fiber strain than neutral and plantarflexed positions. Sarcomere length extracted from muscle fiber length at each ankle angle was used to track the location on the Force-Length curve and showed the MG operates on the curve’s ascending limb. Muscle force changes predicted from the F-L curve going from dorsi-to plantarflexion was less than that experimentally observed suggesting other determinants of force changes with ankle position.

## Introduction

Dynamic studies of isometric and plantarflexion contraction in skeletal muscle using cine-MRI or velocity-encoded phase contrast (VE-PC) MRI have revealed several aspects of muscle deformation that require further exploration [1–4]. The muscle force-length (F-L) relationship is well established and describes the dependence of the steady-state isometric force of a muscle (fiber, or sarcomere) as a function of muscle (fiber, sarcomere) length and it has been explained by the ‘sliding filament’ theory [5–7]. In this theory, the maximal isometric force of a sarcomere is determined by the amount of overlap between the contractile filaments, actin, and myosin [6]. At short lengths, force increases as sarcomere length increases (ascending slope), reaches a plateau at intermediate lengths (optimal length for maximum force production), followed by a decrease in force as sarcomere length increases (descending slope) at long muscle lengths. Muscle fiber architecture (fiber length and pennation angle) clearly influences force production. A study of this structure-function relationship *in-vivo* will reveal aspects of force production that can be used to understand muscle physiology in normal and in diseased states and to develop optimal exercise paradigms for rehabilitation or to maximize athletic performance [8–10]. The medial gastrocnemius (MG) force-length relationship can be altered by changing the knee joint position, ankle joint position, or both. The gastrocnemius muscle is biarticular and spans both the knee and ankle joints; thus, changes in one or both joint angles impact the resting MG muscle fiber architecture (length and pennation angle) and consequently, the force produced by the MG. Several groups have examined the force produced by the MG during isometric plantarflexion contraction for combinations of knee flexion and ankle positions [11–14]. Prior studies have used electromyography (EMG) and ultrasound (US) to study muscle isometric plantarflexion force, activation, and muscle fiber architecture changes in the MG for combinations of knee flexion and ankle angles [11–14]. These studies showed that while there were significant differences in fascicle length of the MG at rest for the different knee/ankle positions, these differences in length were not seen at a maximal isometric plantar flexion contraction (100% Maximum Voluntary Contraction (MVC)) and the EMG activity of the biarticular MG during the MVC decreased at a pronounced flexed knee-joint position despite the fact there were no differences in MG fascicle length at maximum plantarflexion contraction [12]. The authors concluded that the decrease in EMG activity of the MG at pronounced knee flexed positions is due to a critical force–length potential of all three muscles of the triceps surae [12].

The force produced by contracting muscle fibers is transmitted to bones via two passive structures: the aponeurosis and the tendon. These tendinous tissues play an important role as series-elastic-components and can store elastic energy during the movement [3, 15, 16]. The VE-PC technique showed that the strain was heterogeneous with both the deep and superficial aponeurosis exhibiting positive and negative strains along the muscle length [3]. The authors hypothesized that the observed aponeuroses strain may be linked to the distribution and orientation of the forces generated by the muscle fibers [3]. The heterogeneity of fiber length and pennation angle in the proximo-distal direction of the MG may cause nonuniformity of fiber shortening with corresponding changes in regional aponeurosis strain. It is thus likely that, since varying the ankle angle leads to different muscle fiber architecture, it will not only affect muscle fiber strain but also the associated aponeuroses strains. Earlier studies focused on EMG activity and fiber lengths in the MG at rest and during activity at different knee flexion and ankle angles but did not measure muscle fiber and aponeurosis strain at varying ankle angles [12–14]. Velocity-encoded Phase-contrast (VE-PC) MR imaging has been successfully implemented to study muscle kinematics under different contraction paradigms [3, 4, 17, 18]. In the VE-PC method, bipolar magnetic field gradients are applied along one or more spatial directions sensitizing moving spins while refocusing static spins. The resulting phase images of this sequence are directly proportional to the velocity of the moving spins and indirectly proportional to the magnitude of the bipolar gradient. The final phase images are processed to directly provide the velocity values of the moving spins. With the appropriate choice of the bipolar gradient strength to sensitize to the anticipated range of muscle velocities during a dynamic paradigm, the VE-PC method can be ideally customized to the measurement of muscle fiber and aponeurosis motion. The current study focuses on determining the strain in the MG muscle fiber and in the deep and superficial aponeuroses at different ankle angles and with different loads. Dynamic images of the calf muscle were acquired with the foot positioned at plantarflexed, neutral, and dorsiflexed ankle angles and at two different loads corresponding to 25% and 50% of maximum voluntary contraction (MVC), with MVC determined at each foot position. Further, earlier work using dynamic MRI identified the muscle fiber direction by the fascicles on water suppressed images [4]. The fascicles are visualized as higher intensity on the water suppressed images due to the presence of fat deposited adjacent to the fascicles. However, especially in younger subjects, the fascicles are not always visualized consistently since there is little or no fat adjacent to the fascicles in these subjects. The current paper explores an alternate way to extract regional muscle fiber direction using diffusion tensor imaging (DTI) without the additional complexity of fiber tractography. Diffusion tensor imaging provides information on the underlying anisotropy in tissue microstructure and has been extensively applied to map white matter fibers in the brain as well as skeletal and cardiac muscle fibers [19, 20]. DTI derived fiber architecture have been validated by comparison to manual digitization of the same muscle [21]. Of relevance to the current paper, DTI provides the direction of muscle fibers at the voxel level and this feature is used in the current paper to extract the average fiber direction in a regions-of-interest of the MG. The hypotheses are (i) that MG fiber strain will be lowest in the dorsiflexed ankle position while producing the largest force at this ankle angle, (ii) fiber strains will deviate the most from linearity with %MVC in the plantarflexed position, and (iii) MG aponeurosis strain patterns will vary with the ankle angle reflecting the influence of fiber architecture.

## Methods

### Subjects

The study was approved by the Medical Research Ethics Board of University of California at San Diego (UCSD) and conformed to the standards in the Declaration of Helsinki on the use of human subjects in research. All subjects were included in this study after obtaining informed consent. Six healthy, moderately active, male subjects were examined in this study, age: 33.2 ± 16.3 yrs. (range 24–66 yrs.), height: 172.5 ± 7.0 cm (range 163–180 cm), mass: 73.3 ± 6.5 kg (range 63–82 kg). Subjects were excluded if they were involved in vigorous physical training at the level of competitive athletics for the previous 3 months and were also asked to refrain from strenuous activities/ exercise a few days prior to the imaging study.

### MR Imaging

MR imaging was performed on a 1.5 Tesla Signa HDx MR scanner (GE Medical Systems, Milwaukee, WI) with an 8-Ch cardiac coil; the latter was necessary to accommodate the large FOV (30 cm) required to cover the MG from the proximal to distal end. Imaging was performed with the subject lying supine, feet first, with the dominant leg secured in a foot pedal fixture. The fixture allowed for the foot to be positioned at three nominal ankle angles: dorsiflexion (D) 5°, neutral (N) −25°, and plantarflexion (P) −40°. A large FOV image that included the ankle was collected at each foot position using the body coil to verify/estimate the ankle angle. High-resolution water saturated fast-spin echo (echo time (TE): 12.9 ms, repetition time (TR): 925 ms, Echo Train Length (ETL): 7, signal averages (NEX): 4, slice thickness/gap: 3/0 mm, field of view FOV: 30 × 22.5 cm^2^, matrix: 512 × 384) oblique sagittal slices of the calf muscle were collected where water signal is suppressed while the fascicles (fat) appear hyperintense. The slice with greatest fascicle visibility was selected for Velocity-Encoded Phase Contrast (VE-PC) imaging (SS with more than 24 years of experience with VE-PC imaging of the plantarflexors selected the slice for dynamic imaging).

The dynamic gated VE-PC images were collected for a single oblique sagittal slice (TE: 7.7 ms, TR: 16.4 ms, signal averages (NEX): 2, flip angle (FA): 20°, slice thickness: 5 mm, field of view FOV: 30 × 22.5 cm, partial-phase FOV: 0.55, matrix: 256 × 192, gated 22 phases, 3-direction velocity encoding with venc: 10 cm/s, 53 repetitions [192 (phase encode lines) × 0.55 (partial FOV) × 2 (NEX) / 4 (views per segment) = 53]) of 3 second isometric contraction cycles with total scan time 2 min and 39 sec. Force exerted by the subject during isometric contraction was detected by a strain sensor embedded in the foot-plate. Subjects were provided real-time visual feedback of the force generated superposed on the target force curve to facilitate consistent contractions. The differentiated force signal acted as the trigger for gated VE-PC image acquisition. Diffusion Tensor Imaging (DTI) images were acquired anatomically and geometrically matched slice to the VE-PC slice, using a SE-EPI DTI (TE/TR = 63 / 2200ms) sequence with 32 diffusion gradient directions. DTI images were acquired for each ankle angle and corrected for eddy current artifacts, denoised [22], and processed for the eigenvalues/ eigenvectors and fractional anisotropy.

### Force Measurements

The foot pedal’s embedded strain sensor measurements were transmitted via optical fiber cable and recorded by a Data Acquisition device (National Instruments, TX, USA) connected to the computer. Maximum Voluntary Contraction (MVC) was measured for each subject at each ankle angle as the best of three trials recorded prior to imaging: MVC_D_ = 271 ± 49N, MVC_N_ = 140 ± 29N, MVC_P_ = 66 ± 20 N (average over all 6 subjects). The MVCs were significantly different between the three ankle angles: MVC_D−N_ (*p* = 0.0012), MVC_N−P_ (*p* = 0.0003), and MVC_D−P_ (*p* = 0.0012), where the subscripts are the two ankle angles compared in paired t-tests. VE-PC images were collected for submaximal contraction targets of 25% and 50% MVC.

### Muscle Fiber Identification

The deep and superficial aponeurosis of the MG muscle was manually identified (~ 30 seconds) on the VE-PC magnitude image while the rest of the muscle fiber identification process, described below, was automated. The in-house developed algorithm segmented the MG into three regions corresponding to top third (proximal), middle third (middle) and lower third (distal) of its total length. Each region was eroded (3×3structuring element) to be well within the aponeurosis and filtered to remove all voxels with a Fractional Anisotropy less than 0.15 (to exclude noise, fat, and other non-contractile voxels). Since the DTI eigenvectors are 180° indeterminate, the lead eigenvector at each voxel was aligned to point in the same quadrant. The average of the lead eigenvector of each voxel in the region was computed. A line with the average in-plane direction of each region was placed in each region’s center; the ends of this line were extended to intersect the superficial and deep aponeurosis of the MG. This line was designated as the representative fiber direction for the region; a ‘fiber direction’ was identified for each of the three regions. It should be noted that the out-plane component of the lead DTI eigenvector was small, confirming the orientation of the oblique sagittal image captured the MG fibers in-plane of the slice. The DTI out-plane components of the lead DTI eigenvector averaged over the six subjects were small: 8.4%, 9.4% and 7.9% for dorsiflexion, neutral, and plantar flexion positions, respectively. The process of fiber identification for all ankle angles per subject took about three minutes including the manual identification of the aponeuroses of the MG.

### Fiber Strains and Pennation angle

Phase-contrast images were corrected for phase shading artifacts and denoised using a 2D anisotropic diffusion filter [4]. The endpoints of the DTI-identified muscle fibers were tracked through each frame of the dynamic study using the velocity data. It should be noted that DTI data was obtained at rest only. The muscle fiber end points were identified on the DTI images obtained at rest that corresponded to the first frame of the dynamic VE-PC sequence (images were acquired in the contraction cycle with the first frame starting from rest). The muscle fiber in subsequent frames of the dynamic sequence was identified by tracking the fiber end points from the rest frame using the velocity data. Fiber angles were measured with respect to the y-axis of the image (SI direction). Changes in fiber angle were calculated from the initial angle of the fiber. Changes in fiber length were calculated with respect to the initial length and Lagrangian strains were computed. In addition, fiber strains normalized to force were also computed. Strain, changes in fiber length and angle used in the statistical analysis were computed at the peak of the force curve. The fiber strains and pennation angle analysis is completely automated and was completed in less than 30 seconds per subject.

### Aponeurosis Analysis

The deep and superficial MG aponeuroses were manually identified on the first frame of the magnitude VE-PC image for each subject. Each aponeurosis was divided into 11 equal length segments starting from the proximal end of the tibia to the distal end of the MG and tracked through the frames of the dynamic study [3]. Care was taken to position the points in the signal bearing region adjacent to the aponeurosis but not on the aponeurosis itself which has no signal intensity. Length and Lagrangian strains were calculated for each aponeurosis segment for all temporal frames for each ankle angle and %MVC. The identification of the aponeuroses was performed by *BC* and verified by *US* and *SS* (12 and 24 years of experience respectively). This step of the analysis including manual deep and superficial aponeurosis identification followed by automated analysis of segmental strain and visualization completed in about 3 minutes per subject.

### Statistical Analyses

The outcome variables of the fiber analysis are: fiber strain, fiber strain normalized to force, fiber length, and pennation angle; the latter two in the rest and peak contraction frames. Normality of data was tested using both the Shapiro-Wilk test and visual inspection of Q-Q plots. Three-way repeated measures analysis of ANOVA showed that there were no significant differences with fiber location (in the three regions: proximal, middle and distal) in any parameter except in the pennation angle. The values were averaged for the three regions in order to decrease the number of independent variables. Fiber strain and normalized fiber strains were normally distributed and for these variables, changes between ankle angles, % MVC as well as potential interaction effects (ankle angle × %MVC), were assessed using two-way repeated measures ANOVAs and in case of significant ANOVA results for the factor ‘ankle angles’, Bonferroni-adjusted post-hoc analyses were performed. When interactions were present, simple main effects were also examined. Muscle fiber length and pennation angles at rest and peak contraction were not distributed normally, so non-parametric testing was used. For these four outcome variables, the related samples test with Friedman’s 2-way ANOVA by ranks with corrections for multiple comparisons was performed for ankle angle. For all tests, the level of significance was set at α = 0.05. Data are reported as mean (SD) for the variables that are normally distributed and as median (interquartile range, IQR) for those not normally distributed. The statistical analyses were carried out in SPSS for Mac OSX (SPSS 28.0.1.1, SPSS Inc., Chicago, IL, USA).

## Results

**Figure 1:**
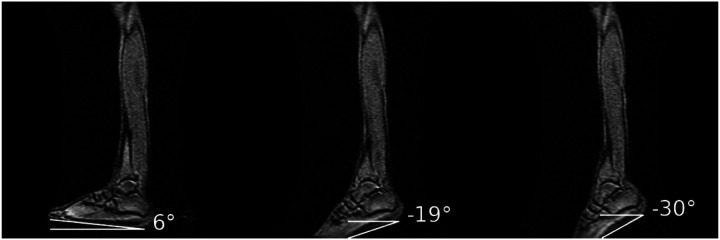
Large field of view (FOV) images for one subject at the three ankle angles (left: dorsiflexed, middle: neutral, right: plantarflexed ankle positions). The images document the actual ankle angle as shown here in the angle measurements at each ankle position.

**Figure 2:**
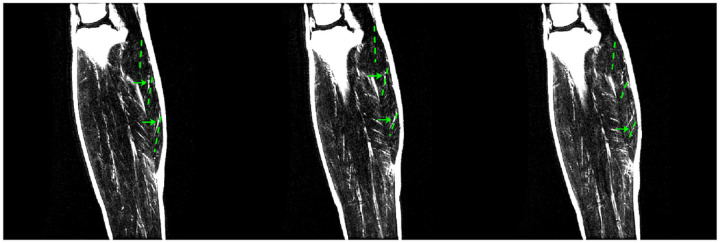
The fibers identified by the proposed method using the DTI lead eigenvector data are shown in green dashed lines in the three regions of the muscle (proximal, middle and distal) superposed on the water saturated Fast Spin Echo images (muscle appears dark while the fascicles due to the presence of fat appear bright on these images). A few fascicles in the MG can be seen on the water saturated images and these are approximately aligned with the DTI derived fibers (identified by green arrows). It can be seen that not many fascicles are visible in the MG while the proposed technique using DTI is effective in identifying the muscle fibers.

Figure 1 shows the ankle angle measurements determined from large FOV sagittal slices. Figure 2 shows the fibers identified by the average of the lead eigenvector method for the proximal, middle, and distal regions of the MG for each % MVC and ankle angle for one subject; the fibers are superposed on high-resolution water saturated fast-spin echo images. The orientation of the fibers is as expected for the MG and follows the direction of fascicles. Supplemental Video V1 shows the video of the fibers with the motion of the fiber end points tracked through the dynamic cycle for one subject for the two %MVCs and at the three ankle angles.

**Figure 3:**
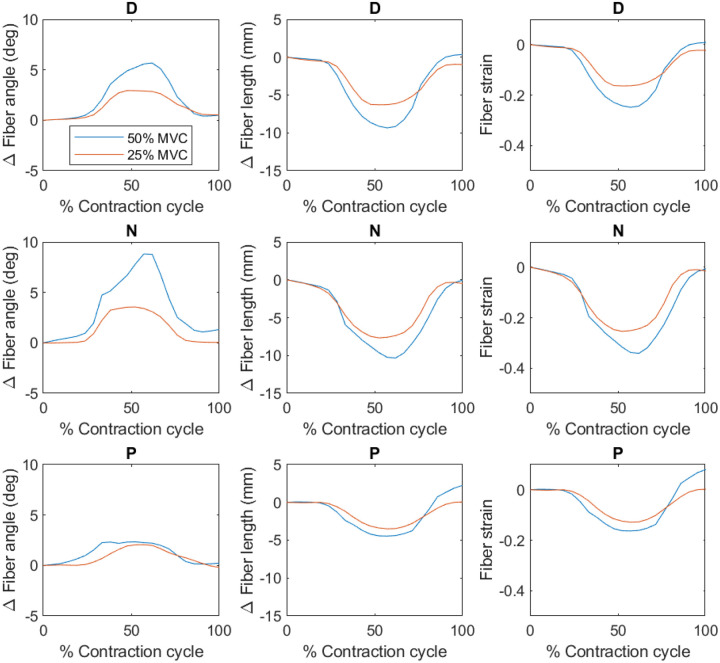
The variation, as a function of the dynamic cycle, of change in fiber angle from the initial frame, change in fiber length from the initial frame and fiber strain is shown for one subject (top row: dorsiflexed (D) ankle position, middle row: neutral (N) ankle position, bottom row: plantarflexed (P) ankle position. The changes with %MVC is the least for the plantarflexed position. The values at the peak of the contraction are reported in Tables 1a and 1b (averaged over the six subjects).

Figure 3 depicts the changes in fiber angle, length and strain as a function of the contraction cycle for one subject. Table 1a lists the mean and standard deviation of MVC, peak force, fiber strain, and normalized fiber strain for each foot position and % MVC. Table 1b lists the median and interquartile range of fiber architecture at rest and at peak contraction. The peak strain was significantly lower at the lower %MVC (*p* = 0.002) and while peak strain was the lowest in the dorsiflexed position, it was not significantly different from other two ankle angles (Table 1a). Further, fiber strain changed significantly between 25 and 50%MVCs in the dorsiflexed (*p* = 0.004) and neutral (*p* = 0.034) positions but not in the plantarflexed position (Table 1a). As (ankle angle * force) interaction was significant for strain normalized to force, simple main effects are reported for strain normalized to force. At both 50%MVC and 25% MVC, strain normalized to force was significantly different between each pairwise combination of ankle angles (Table 1a). At 50%MVC the p-values for the differences in normalized strain between ankle angles were the following: D-N (*p* = 0.022), D-P (*p* = 0.004), N-P (*p* = 0.012) and corresponding values at 25%MVC were *p* = 0.037, 0.012, 0.016 respectively. The absolute value of the normalized strain was lowest at the dorsiflexed position at 50% MVC while the highest normalized strain was at the plantarflexed position at 25% MVC (Table 1a). Comparing the normalized strain at 50 and 25% MVC at each ankle angle showed that significant changes with %MVC was only seen in the P ankle position (*p* = 0.029). The absolute value of normalized strain at 25% MVC was significantly higher than at 50% MVC for the P ankle angle. Significant differences were seen in the resting fiber length between D-P (*p* < 0.001) and D-N (*p* = 0.023) and in resting pennation angle between D-P (*p* = 0.029) while a trend was observed in D-N (*p* = 0.059) and N-P (*p* = 0.059) (Table 1b). Resting fiber length decreased and pennation angle increased going from D to P (Table 1b). Significant differences in fiber length at peak contraction were seen with force (*p* < 0.001) and with ankle angle: D-P (*p* < 0.001) and D-N (*p* < 0.001). Significant differences in pennation angle at peak contraction were seen with force (*p* < 0.001) and with ankle angle: D-N (*p* = 0.029) and D-P (*p* < 0.001). At peak contraction, fiber lengths decreased and pennation angles increased compared to corresponding values at rest.

**Figure 4:**
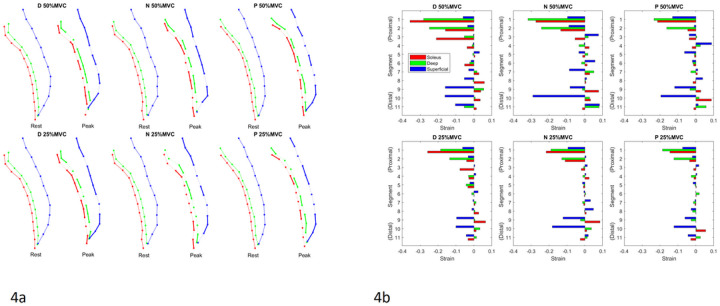
4a: The end points of the 11 segments are shown for the medial gastrocnemius deep (green) and superficial (blue) aponeuroses. The points on the soleus to track the aponeurosis are shown in red. The top row shows 50% MVC while the bottom row shows 25% MVC. The schematic is presented in pairs (at rest and at peak contractions) from left to right: dorsiflexed (D), neutral (N), plantarflexed (P) ankle angles. In the paired schematics, the segments at the moment of peak contraction are shown as shortening (thick solid line), expanding (dotted line) or unchanged (thin solid line). 4b: Plot of the segmental strain values extracted at peak of the dynamic contraction for 50% MVC (top row) and for 25% MVC (bottom row) for dorsiflexion (D), Neutral (N) and plantarflexion (P) ankle angle positions. Segment 1 is the proximal end while segment 11 is the distal end of the aponeuroses, the segments are arranged vertically starting from distal (lower end) to proximal end (top) so that it aligns with the segments shown in Fig. 4a. Each segment has three bar plots corresponding to red (Soleus), green (deep aponeurosis), blue (superficial aponeurosis); there are eleven sets with three bar plots each corresponding to the eleven segments in Fig. 4a. The close match of the segments tracked from the soleus and medial gastrocnemius sides of the distal aponeurosis (red and green, respectively) is a check of the internal consistency of the velocity-based tracking.

Figure 4a shows, for one subject, the schematic of the locations of the 11 points placed along the MG deep and superficial aponeuroses and the line segments connecting them at rest and at peak contraction. Figure 4b is box plot of the aponeurosis strain (estimated at peak force) in the 11 segments for the two %MVC and the three ankle angles averaged over the six subjects. Analysis of the strain comparing corresponding segments revealed no significant differences between the three ankle angles. Across all three ankle angles, aponeurosis strain was high at the distal and proximal regions of the muscle length and lowest in the middle where it was close to zero strain. The distal end of the deep aponeurosis showed small positive strains whereas the superficial aponeurosis revealed negative strains at the distal end. Further, for the superficial aponeurosis, the absolute value of strains at the distal segments were the highest among all the segments. The deep aponeurosis showed the highest negative strains in the proximal end, higher than strain values in the superficial aponeurosis. The motion of the segments along the aponeurosis during the contraction cycle is shown in Supplemental Video V2.

**Figure 5:**
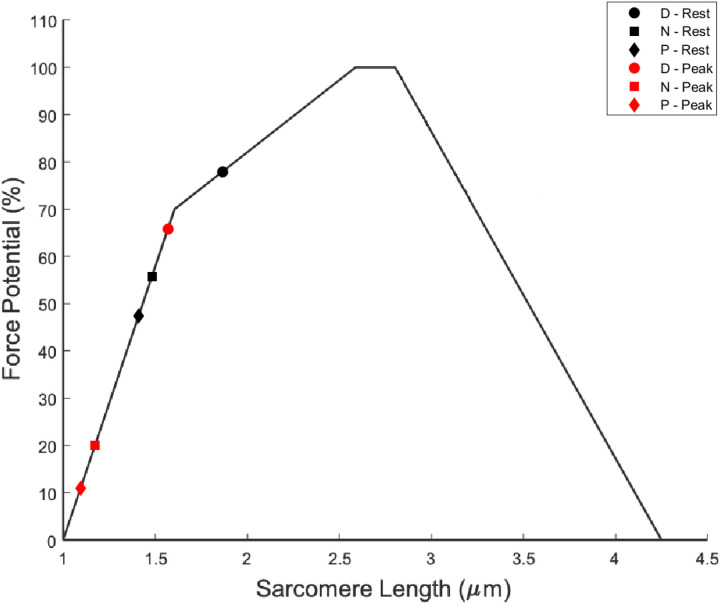
The Force- Length curve (solid line) is plotted based on data from Ref. 24. The black markers pertain to the sarcomere length calculated for the resting fiber length at each ankle angle (Dorsiflexion: D, Neutral: N, Plantar flexion: P) while the red markers are calculations made with lengths at peak contraction at 50% MVC (Table 2). All values were calculated using average values of fiber lengths for the six subjects. An initial estimate of 1.9 microns for the dorsiflexed position was made to approximately follow the experimentally observed changes in force with ankle angle.

An exploratory analysis was also conducted to determine if the changes in MVC with ankle angle can be explained by the relative positions of the muscle (sarcomere) length on the F-L curve. To this end, the relative sarcomere length at different ankle angles was computed assuming a reference sarcomere length for the dorsiflexion ankle position. The reference value was chosen such that it was closer to the optimal sarcomere length for maximum force (on the F-L curve) since the dorsiflexor position generated the maximum force (compared to the other two ankle positions). A sarcomere length of 1.9 μm was assumed for the dorsiflexion ankle position; the choice of this reference sarcomere length in the D position was made to approximately follow the changes in MVC determined experimentally when going from D to N to P. For example, choosing a sarcomere length closer to the peak of the F-L curve (2.5 microns) resulted in much smaller changes in MVC as a function of the ankle angle than observed experimentally. Assuming a sarcomere length of 1.9 um for the D position and with the measured fiber length, the number of sarcomeres was computed. As the number of sarcomeres is not expected to change with ankle angle, the same sarcomere number was used to calculate the sarcomere length for muscle fiber length at rest and at 50% MVC (Table 2). Figure 5 shows the sarcomere lengths calculated from muscle fiber lengths at rest (black) and at 50%MVC (red) superposed on theoretical F-L relationship which was derived from data reported in [23, 24]. The rationale behind generating the plot shown in Fig. 5 is that it allows one to estimate the change in MVC (with ankle angle position) from the F-L relationship since muscle fiber length (and consequently, the sarcomere length) changes with ankle angle. The plot shows the MVC that can be attained for muscle fiber lengths measured at rest for the three ankle angles and provides a qualitative explanation for the decrease in MVC when the ankle angle changes from D to N to P.

## Discussion

The specific objectives of this study are to implement a diffusion tensor imaging method to identify MG fibers and to map fiber strains using VE-PC MRI for submaximal isometric contraction with ankle angle held in dorsiflexion, neutral, and plantarflexion angles. The hypotheses are (i) that MG fiber strain will be lowest in the dorsiflexed ankle position while producing the largest force at this ankle angle, (ii) fiber strains will deviate the most from linearity with %MVC in the plantarflexed position, and (iii) MG aponeurosis strain patterns will vary with the ankle angle reflecting the influence of fiber architecture.

Prior work used fascicles that were manually identified on water saturated anatomical images and the end points of these manually identified fibers were tracked through the dynamic cycle using the VE-PC data [4]. This method is limited in utility due to the difficulty of identifying fascicles especially in young subjects. The reason that fascicles have high contrast on water saturated images is the presence of fat adjacent to the fascicles; however, the fat layer is minimal in younger subjects making it difficult to view the fascicles. The method proposed here to identify fiber directions from the lead eigenvector of the DTI data is shown to be feasible. It should be noted that the proposed method does not involve muscle fiber tractography which would provide the most accurate estimate of the muscle fibers. However, accurate and robust fiber tracking requires DTI data with the highest SNR (single voxel accuracy of the lead eigenvector for fiber tracking). In contrast, the SNR requirements to accurately determine the average of the lead eigenvector in a ROI (27 pixels are higher) are lower [25]. The proposed method thus allows for short DTI acquisition times since it uses average data over fairly large ROIs (> 27 pixels). It should also be noted that a DTI scan is acquired at each ankle angle, so minimizing the acquisition time for DTI is important; each DTI scan is completed in ~ 7 minutes in the current work. The DTI scans also confirmed that the method for selecting the oblique sagittal slice yielded MG fibers primarily in the plane of the image.

The significant changes in MVC with ankle angle position in isometric contractions seen in the current study have been reported in earlier studies [12]. The dependence of the MG MVC on the ankle angle position is attributed to ankle dorsiflexion stretching the gastrocnemius and bringing it closer to its optimal length in the force-length curve, which contributes to knee flexion strength and knee joint stability, thereby enabling greater muscle force [12]. In all ankle positions, as anticipated, fiber length decreased and pennation angle increased at peak contraction compared to the values at rest. The current study also confirms US studies that showed fiber lengths and pennation angles to be significantly different between ankle positions at rest [12]. However, the current study also shows that significant changes in fiber length and pennation angle with ankle angle (between D-N and D-P) persisted at peak contraction contrary to US studies that found no significant differences in the fiber lengths and pennation angles at peak force [12]. This may reflect the fact that US studies were at 100% MVC whereas the current study was at submaximal isometric contractions (25 and 50% MVC).

EMG activity of the MG has been reported to decrease during MVC for knee flexed positions and ankle angles at the more plantarflexed position [12]. The main mechanism postulated in Ref. 12 for decreasing EMG activity and impairment in neuromuscular transmission-propagation at short muscle lengths is a neural inhibition. This is hypothesized as being triggered as the muscle reaches a critical shortened length at which, due to the force-length relationship, the torque output cannot be increased even if the muscle is fully activated. The human control system gets feedback regarding the force potential due to the force-length relationship of all three muscles of the triceps surae and regulates their activity to increase the economy of maximal torque generation [12]. In contrast to the decrease in EMG activity at the more knee flexed/ plantarflexed ankle positions [12], fiber strain is higher at the P ankle position compared to the D ankle position in the current study (while MVC at D ankle angle MVC was higher than the MVC at P ankle angle). This points to the fact that the MG is inefficient in producing force in the plantarflexed ankle position; i.e., despite a larger contraction (strain) at the plantarflexed ankle position the force produced is smaller. The fact that the MG did not show decreased strains at the P ankle angle when going from 25 to 50% MVC indicates that it has not yet reached actively insufficiency at the P ankle angles of + 30° and submaximal isometric contraction used in the current study.

Fiber strains in the MG were lower (though it did not reach significance) in the D ankle position compared to the P and N position and significantly higher at 50% MVC than at 25% MVC in the D and N positions. The increased strain at higher %MVC in the D and N ankle positions is understandable as increased contraction is required to generate the higher forces. In contrast to D and N, the observation that the P ankle position did not show significant change in fiber strain with %MVC may indicate that the MG may be approaching the critical length where further contraction becomes more difficult. It is surprising that the fiber strains are lower in the D ankle position since in this ankle position, higher force is generated. It would be expected that the higher force in the D ankle angle will be accompanied by larger strains; the implication of the lower strains is that the D ankle angle position is ideal for force generation in that even small contractions (strains) are sufficient to generate large forces. Fiber strains normalized to force showed significant differences with ankle positions at both force levels. In comparing ankle positions, the D ankle position showed significantly lower normalized strains at both %MVCs than the N and P ankle positions; highlighting the fact that the D ankle position is at the optimum position for force production followed by N and the least optimal for the P ankle position. Smaller strains (contractions) are required to generate the same force at the dorsiflexed position than in the other two positions. Further, the absolute value of the normalized fiber strain was significantly higher at 25%MVC than at 50% MVC for the plantarflexed position. The latter finding is a reflection of strain not increasing significantly with %MVC in the P ankle position. It is likely that compared to the D and N positions, the contribution of the soleus to the total force increases in the P ankle position at higher %MVCs. The finding of lower strain and lower normalized strains in the dorsiflexed position while generating a high force should be of interest in rehabilitation paradigms, in optimizing athletic performance, and in minimizing strain injuries [26].

In terms of the aponeurosis strains during isometric contraction, ultrasound studies showed that the superficial and deep aponeuroses of medial gastrocnemius (MG) are uniformly stretched along their lengths in opposite directions; the superficial aponeurosis is stretched distally, whereas the deep aponeurosis is stretched proximally [27]. However, a study by Kinugasa *et al*. using the MR VE-PC technique revealed heterogeneous aponeurosis strain patterns: positive strain occurred at both ends (proximal and distal) of the deep aponeurosis and in the proximal region of the superficial aponeurosis while negative strain was observed in the middle region of the deep aponeurosis and in the distal region of the superficial aponeurosis [3]. Similar patterns of aponeurosis heterogeneity are also seen in the current study as in the earlier MR paper [3] if it is noted that the proximal most position of the current study is the third segment of the earlier study by Kinugasa *et al*. (the latter study started the segments at the distal end of the femur while the current study starts at the proximal end the tibia). Thus, the positive strain that was seen in the first two proximal segments of the deep and superficial aponeuroses in the earlier study is not seen in the current study. Contrary to the hypothesis, the strain patterns did not show significant differences between the three ankle positions.

The length of sarcomeres that are arranged in series within a striated muscle fiber is one of the most important determinants of muscle force [23]. As the ankle angle changes, the fibers of the MG and their constituent sarcomeres change length, which shifts the sarcomeres’ positions on the sarcomere force–length curve and affects the force-generating capacity of the muscle. It should be noted that the current study does not measure sarcomere length directly at any of the ankle angles but given the highest value of force at dorsiflexion position, this ankle position was assumed to closer to the optimum sarcomere length than the N or P positions. Further, once the sarcomere length for the dorsiflexion ankle angle position is chosen close to the optimum sarcomere length of the F-L curve, the current study shows that the sarcomere lengths of the MG at the other two ankle angles are positioned on the ascending limb of the F-L curve. This has also been observed in ultrasound and dynamometry studies of the soleus where it was shown that soleus acts on the ascending limb during active contractions [28]. It should be noted that the choice of the reference sarcomere length at 1.9 μm for the D position was dictated by considerations that the calculated sarcomere lengths at other ankle angles resulted in force decreases on the F-L curve close to that observed experimentally. Based on the F-L curve, the choice of the optimal sarcomere length of 2.65 μm (generates maximum force) severely underestimated the observed force decrease with ankle angle. In fact, even the reference value of 1.9 μm underestimates the force decreases with ankle angles.

These results potentially indicate that there may be other determinants of the observed force changes with ankle angle beyond changes in the sarcomere length. As the aponeurosis strains are not different between the ankle angles, this is not likely to contribute to the observed force changes. It should also be noted that while an isometric contraction implies no change in muscle length, it does not mean imply an unchanged muscle fiber length. As seen experimentally, muscle fiber contracts and rotates leading to sarcomere lengths at peak contraction shifting further down the ascending limb of the F-L curve. It is possible that the force potential is determined by an average of the rest and peak sarcomere length than just the rest sarcomere length. It is also likely that factors other than changes in sarcomere length may contribute to the measured force changes. It is important to emphasize that while the current method does not provide absolute values of sarcomere length, it allows relative changes in sarcomere length with ankle position to be estimated. That is, by fixing the sarcomere length at one reference point (the dorsiflexed ankle angle in the current study) one can estimate sarcomere lengths obtained from experimentally observed fiber lengths at rest and at peak contraction as a function of ankle position, and map these lengths on the F-L curve to determine whether the muscle is operating on the descending or the ascending limb of the force-length curve. The current study is focused on establishing the technical feasibility of muscle strain mapping combining DTI and velocity encoded dynamic data to determine the relationship of force and fiber strain to fiber architectural differences simulated by different ankle angles. Further studies are required to extend this to fielderly participants or patients with specific conditions, such as lower extremity paralysis or muscle rigidity following stroke. For example, muscle contractures that result from upper motor lesion in patients with cerebral palsy are often treated by surgical lengthening [29]. Mathewson *et al*. have posited that it is important to know both fascicle length and sarcomere length to make appropriate intraoperative surgical decisions about muscle lengthening; the current study can provide information on whether the sarcomere length is on the ascending or descending limb of the F-L curve [30].

There are some limitations to the current study, the main one is the small sample size. However, it should be noted that the repeated measures ANOVA has higher statistical power since the variability in the subject population is taken into account. Further, statistically significant differences in fiber strain, normalized fiber strains and in fiber architecture with force and ankle angles were identified in the current small study. The study was in 2D while true muscle fibers traverse a 3Dal space both at rest and at the peak of the contraction. However, care was taken to position the oblique sagittal slice such that the fibers of the MG lay in the plane of the image; this was subsequently verified by the DTI based analysis which showed that the fibers ran predominantly in-plane. It should also be noted that the 2D acquisition allowed for the MG fibers to be in the imaging plane but this plane is not necessarily in the right orientation to capture the soleus fibers in the imaging plane. Further, the more complicated orientation of the soleus muscle as well as its sub-compartments will make it near impossible to capture the fibers of the entire soleus muscle in a single plane. A 3D, three directional velocity encoding sequence is required to map fiber strains in all the plantarflexor muscles.

In summary, the current paper focuses on muscle fiber and aponeurosis strains obtained at two levels of submaximal isometric contractions with the ankle angle varied from dorsiflexion to neutral to plantarflexed. The dorsiflexed ankle position generated significantly greater force while exhibiting significantly lower normalized strains than the neutral or plantarflexed position. The sarcomere lengths at rest and at peak force were calculated assuming a reference optimal sarcomere length at the dorsiflexed ankle angle and the MG was identified to be working in the ascending limb of the FL curve. The analysis of the sarcomere lengths and their relative positions on the F-L curve also revealed that there may be other determinants to force changes with ankle position in addition to changes in sarcomere length.

## Figures and Tables

**Table 1a:** Muscle fiber strain and fiber strain normalized to force.

Ankle Position	% MVC	MVC (N)	Peak Force (N)	^1d^ Peak Strain	^1a, 1b, 1c, 1d, 2a, 2b, 2c, 2d^ Strain / Force (1/N)
D	50%	271.0 [48.7]	131.0 [31.1]	−0.1545 [0.0656]	−0.0013 [0.0007]
D	25%	271.0 [48.7]	68.5 [15.7]	−0.1029 [0.0466]	−0.0015 [0.0007]
N	50%	140.2 [29.2]	71.9 [20.1]	−0.2179 [0.0945]	−0.0032 [0.0016]
N	25%	140.2 [29.2]	36.2 [10.4]	−0.1539 [0.0774]	−0.0045 [0.0025]
P	50%	66.4 [20.5]	31.6 [13.9]	−0.2084 [0.0730]	−0.0076 [0.0036]
P	25%	66.4 [20.5]	16.4 [7.4]	−0.1742 [0.0694]	−0.0123 [0.0061]

Note: Ankle positions are dorsiflexion (D), neutral (N), and plantar flexion (P).

Standard deviation values are provided in square brackets beneath mean values.

1a 50% MVC: Significant difference between ankle positions D and N

1b 50% MVC: Significant difference between ankle positions N and P

1c 50% MVC: Significant difference between ankle positions D and P

1d D and N: Significant difference between 25% and 50% MVC

2a 25% MVC: Significant difference between ankle positions D and N

2b 25% MVC: Significant difference between ankle positions N and P

2c 25% MVC: Significant difference between ankle positions D and P

2d P ankle angle: Significant difference between 25% and 50% MVC

**Table 1b:** Muscle fiber architecture at rest and at peak contraction.

Ankle Position	MVC	^c^ Rest Angle (°)	^a, c, d^ Peak Contr.* Angle (°)	^a, c^ Rest Length (mm)	^a, c, d^ Peak Contr.* Length (mm)
D	50%	32.2 [1.5]	34.1 [3.3]	42.6 [13.6]	35.9 [11.4]
D	25%	32.2 [1.5]	33.4 [4.6]	42.6 [13.6]	38.8 [13.0]
N	50%	33.3 [3.5]	34.6 [7.3]	34.5 [11.8]	28.2 [10.7]
N	25%	33.3 [3.5]	33.2 [5.3]	34.5 [11.8]	32.0 [13.8]
P	50%	35.1 [4.3]	37.3 [5.7]	29.0 [5.8]	23.0 [6.2]
P	25%	35.1 [4.3]	36.6 [5.6]	29.0 [5.8]	25.2 [5.2]

Note: Ankle positions are dorsiflexion (D), neutral (N), and plantar flexion (P).

Interquartile range is provided in square brackets beneath median values.

* Contr.: abbreviation for contraction.

a significant difference between ankle positions D and N.

b significant difference between ankle positions N and P.

c significant difference between ankle positions D and P.

d significant difference between 25% and 50% of maximum voluntary contraction (MVC).

**Table 2:** Muscle fiber length and sarcomere length at rest and at peak contraction.

Ankle Position	Number of Sarcomeres	Rest Sarcomere Length (μm)	Rest Fiber Length (mm)	Peak Contr.* Sarcomere Length (μm)	Peak Contr.* Fiber Length (mm)
D	24526	1.9	46.6	1.6	39.8
N	24526	1.5	37.6	1.2	29.9
P	24526	1.4	33.2	1.1	26.6

Note: Ankle positions are dorsiflexion (D), neutral (N), and plantar flexion (P).

* Contr.: abbreviation for contraction.

## Data Availability

The datasets analyzed during the current study are available from the corresponding author upon reasonable request.
